# AI in daily cardiology and structured multidisciplinary care

**DOI:** 10.1007/s12471-026-02094-y

**Published:** 2026-10-15

**Authors:** Pim van der Harst

**Affiliations:** Department of Cardiology, University Medical Centre Groningen, Groningen, The Netherlands

This issue of the Netherlands Heart Journal includes three original articles, Dutch endorsement of the 2025 ESC pregnancy guideline, and a  perspective on artificial intelligence (AI) in the outpatient clinic.

In a national survey among 120 hospital-based professionals, Aertsen-van der Kuip et al. found that 97% considered palliative care and advance care planning in chronic heart failure important, but only one third provided it regularly [1]. While 78% recognised palliative care needs, only 23% felt sufficiently skilled. Education and time remain key barriers.

Van Vreeswijk et al. describe EmbRACE, a national network on atrial cardiomyopathy [2]. Seven work packages combine biobanking, molecular profiling and AI-based modelling with interventional trials. The aim is to define thresholds and determine whether targeting the atrial substrate improves outcomes.

Van der Zwaan et al. present the NVVC endorsement of the 2025 ESC pregnancy guidelines [3]. Women with cardiovascular disease should undergo mWHO 2.0 risk assessment. Women above class II are counselled and followed by a Pregnancy Heart Team. The Dutch addendum includes recommendations on single-embryo transfer and ethical deliberation.

Okken et al. developed an AI tool that detects complications of pulmonary vein isolation, coronary intervention and TAVI from health records [4]. In 6,564 patients, sensitivity was 94.4–100% and specificity 99.4–100%. Flagged cases still require review, but registration no longer relies solely on manual reporting.

Meijer and De Jong describe the Epic-integrated ambient AI scribe at OLVG [5]. They observed more patient attention and about 30% less documentation time, but also errors such as pantoprazole transcribed as “plantenzorg”. AI-generated notes should always be reviewed before signing.

These AI applications can take over part of the administrative work in cardiology, with the clinician still responsible for the output. Wider use will require local validation and clear agreements on who verifies what.
